# A generic strategy for CRISPR-Cas9-mediated gene tagging

**DOI:** 10.1038/ncomms10237

**Published:** 2015-12-17

**Authors:** Daniel H. Lackner, Alexia Carré, Paloma M. Guzzardo, Carina Banning, Ramu Mangena, Tom Henley, Sarah Oberndorfer, Bianca V. Gapp, Sebastian M.B. Nijman, Thijn R. Brummelkamp, Tilmann Bürckstümmer

**Affiliations:** 1 Horizon Genomics, Campus Vienna Biocenter 3, 1030 Vienna, Austria; 2 Horizon Discovery, 7100 Cambridge Research Park, Waterbeach, Cambridge CB25 9TL, UK; 3 Ludwig Cancer Research, Nuffield Department of Clinical Medicine, University of Oxford, Oxford OX3 7DQ, UK; 4 Netherlands Cancer Institute, Plesmanlaan 121, 1066 CX Amsterdam, Netherlands

## Abstract

Genome engineering has been greatly enhanced by the availability of Cas9 endonuclease that can be targeted to almost any genomic locus using so called guide RNAs (gRNAs). However, the introduction of foreign DNA sequences to tag an endogenous gene is still cumbersome as it requires the synthesis or cloning of homology templates. Here we present a strategy that enables the tagging of endogenous loci using one generic donor plasmid. It contains the tag of interest flanked by two gRNA recognition sites that allow excision of the tag from the plasmid. Co-transfection of cells with Cas9, a gRNA specifying the genomic locus of interest, the donor plasmid and a cassette-specific gRNA triggers the insertion of the tag by a homology-independent mechanism. The strategy is efficient and delivers clones that display a predictable integration pattern. As showcases we generated NanoLuc luciferase- and TurboGFP-tagged reporter cell lines.

## Abstract (web summary)

CRISPR-Cas9 has greatly enhanced genome engineering however gene tagging can still be cumbersome due to a requirement for homology donors. Here the authors introduce a generic system for gene tagging that does not require homology between the donor and the genomic target site.

## Graphical abstract

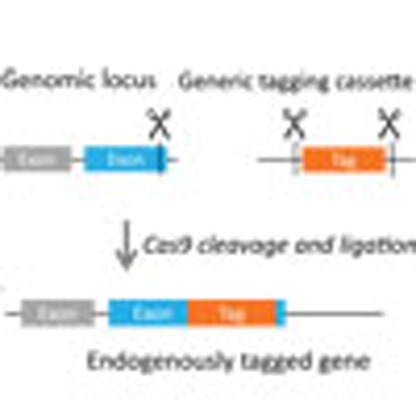

Genome engineering has been revolutionized by the discovery of the CRISPR-Cas9 system. Cas9, a bacterial endonuclease from *Streptococcus pyogenes*, can be programmed by a small guide RNA (gRNA) to trigger a double-strand break at any desired genomic locus that is followed by a protospacer-adjacent motif (PAM, NGG for Cas9 from *Streptococcus pyogenes*)^1^,^2^,^3^,^4^. The ensuing double-strand break can either be repaired by non-homologous end joining (NHEJ), which is often imprecise leading to small insertions or deletions (indels), or by homology-directed repair, if a suitable homology template is present. CRISPR-Cas9 technology has been widely adopted for the rapid generation of gene knockouts in various organisms^5^.

Gene tagging, however—the fusion of endogenous genes to foreign DNA sequences—has remained cumbersome, mostly because it requires the design and creation of a homology template^4^ that is specific for each gene to be tagged. In this template, the foreign sequence is generally flanked by homology arms of 500–1,500 bp on either side that are specific to the targeted endogenous locus. Tagging different genes with an identical tag—for example, green fluorescent protein (GFP)—will thus require the design and synthesis of a separate donor for every gene, which is costly and laborious.

We therefore sought to develop an approach for gene tagging based on CRISPR-Cas9 that alleviates the need for homology templates. Previous reports using zinc-finger nucleases, TAL effector nucleases or CRISPR-Cas9 technology have shown that plasmids containing an endonuclease cleavage site can be integrated in a homology-independent manner^6^,^7^,^8^. While these approaches were generic, that is, did not require any donor adaptation to the locus that was targeted, they led to the integration of the entire donor plasmid, which is often undesired and hampers N-terminal gene tagging. Alternatively, several labs have developed approaches that were based on microhomologies between the donor vector and the target site^7^,^9^. While one of these approaches did not lead to the incorporation of the entire plasmid backbone^9^, it did require adaptation of the donor to the targeted locus and is thus not generic.

The approach we developed had the following four major goals: (i) it should be generic, that is, one donor template could be used for the tagging of any genomic locus; (ii) it should be robust and precise, such that a sequence with defined 5′ and 3′ ends can be integrated in a predictable manner; (iii) it should be efficient, that is, screening a moderate number of clones should be sufficient to isolate a cell line containing the tagged allele; (iv) it should be applicable to human cells.

Here we describe the successful establishment of such a strategy that allowed us to endogenously tag genes at both the N and C terminus, without the integration of adjacent plasmid sequences. While we demonstrate the feasibility of this approach with both a NanoLuc luciferase- and TurboGFP-tagging cassette, this approach can be used to integrate any sequence of choice into the genome. We furthermore show that tagged alleles retain full functionality both in terms of gene expression regulation as well as subcellular localization similarly to the untagged endogenous alleles.

## Results

### A homology-independent approach for gene tagging

**Figure 1:**
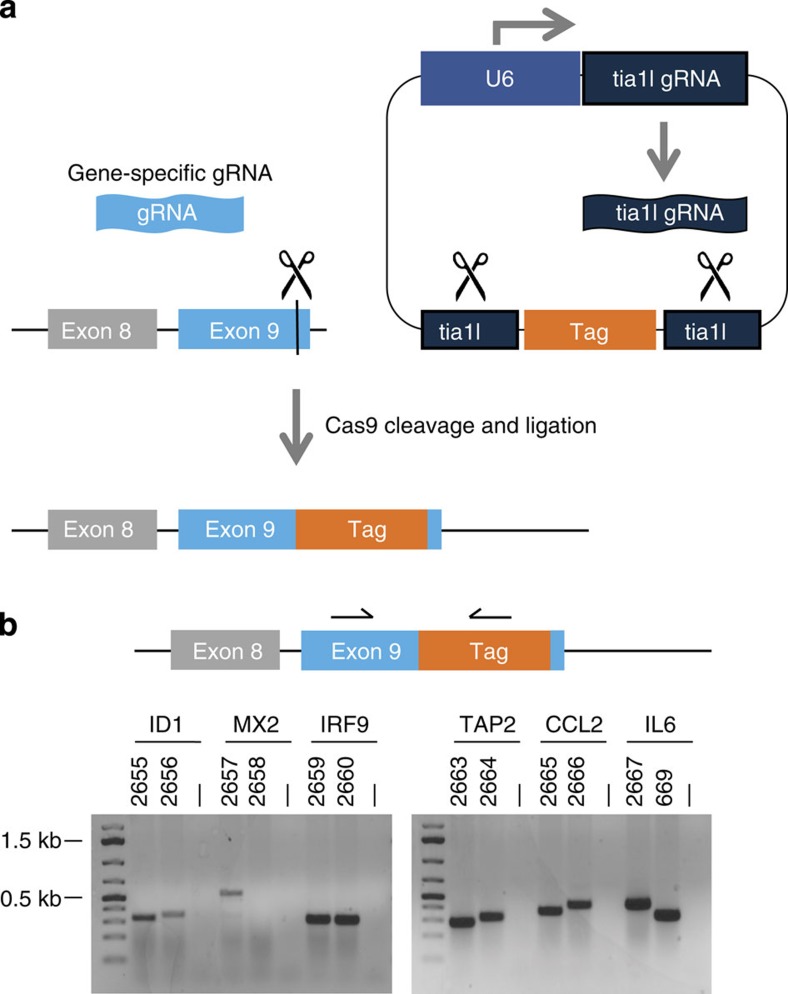
Approach for homology-independent gene tagging. (**a**) Schematic representation: cells are transfected with Cas9, a gRNA specifying the desired locus in the human genome (here in exon 9 of the gene of interest) and a generic donor plasmid. The generic donor plasmid contains the tag of interest, flanked by two tia1l recognition sites, as well as a U6 promoter driving the expression of the tia1l gRNA. Consequently, the tag of interest will be released upon co-expression of Cas9 and spontaneously integrated at the site specified by the genomic gRNA. (**b**) HAP1 cells were transfected with expression plasmids for Cas9, the tia1l gRNA and the generic NanoLuc donor. For each gene under consideration, we chose two independent gene-specific gRNAs that were co-transfected. Genomic DNA was isolated from pools of cells 5 days post transfection and analysed by PCR. For this PCR, one constant primer binding to the NanoLuc cassette (5′-GGATCGGAGTTACGGACACC-3′) was combined with one variable primer for each gene of interest. HAP1 wild-type cells were included as a reference (lanes labelled with —). Numbers above each lane define the guide RNA identity, as specified in Fig. 2 and Table 1.

We designed a generic plasmid donor harbouring the tag of interest (Fig. 1a), which is flanked by two gRNA cleavage sites that correspond to a genomic locus in Zebrafish (tia1l) that is absent in human cells^10^. The plasmid donor also encodes a U6 promoter driving the expression of the tia1l gRNA. When cells are transfected with Cas9, the donor plasmid and a gRNA specific to the region of the gene where the tag should be incorporated, the tag will be released from the plasmid and subsequently integrated into the gene of interest.

To assess whether the donor could be employed to create tagged alleles, we used the human near-haploid cell line HAP1 (refs 11, 12), because it contains a single copy of most genomic loci and is thus ideal to trace genomic-editing events. As an initial proof of concept, we picked six genes and selected two gRNAs targeting the 3′-coding region of each gene. We transfected HAP1 cells with the donor plasmid (Supplementary Fig. 1), expression plasmids for Cas9 and one gRNA specifying the genomic locus of interest and a plasmid encoding a blasticidin resistance gene. We eliminated untransfected cells by applying a short pulse of blasticidin. After selection, we assessed integration of the donor in the pool of transfected cells by a PCR strategy where one primer binds in the genome and another primer binds in the tagging cassette (Fig. 1b). Specific gene-tagging events were evidenced by the occurrence of a specific PCR product in transfected cells that is absent in the parental cell line. A total of 11 out of 12 targeting events showed integration of the donor cassette (Fig. 1b). The failed targeting event was not due to inefficient Cas9 cleavage, as all gRNAs showed comparable efficiencies when evaluated in a T7 endonuclease assay (Supplementary Fig. 2). These initial findings suggest that our approach led to the successful integration of the donor and that our strategy is applicable across diverse genomic loci.

### Gene tagging is efficient and precise

To assess the frequency of integration events, we isolated clonal cell lines by limiting dilution from nine separate gRNA transfections and analysed clones by PCR and Sanger sequencing (Table 1). The results showed that we were able to retrieve clones bearing tagged alleles for five of the nine gRNAs. Importantly, in some cases, we were able to isolate multiple clones from a very moderate total number of 24 clones tested, indicating a high overall efficiency of the procedure.

**Figure 2:**
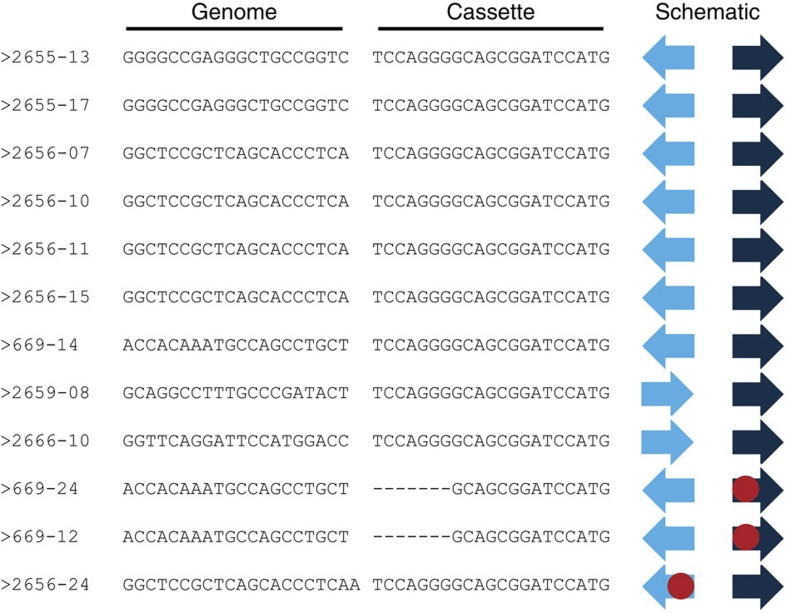
Gene tagging is efficient and precise. Sanger sequencing data of clones described in Table 1 were analysed to identify the integration pattern. Dark-blue arrows indicate the directionality of the tia1l gRNA, light-blue arrows indicate the directionality of the genomic gRNA site. Red dots symbolize additional insertions or deletions identified by Sanger sequencing. Number on the left specify the clone ID.

When analysing the sequences obtained from clonal cell lines bearing tagged alleles, we initially expected to see insertions or deletions as a consequence of the imprecise nature of NHEJ. In contrast, we observed that 9 out of 12 clones showed a perfect cleavage and ligation pattern (Fig. 2): in these clones, Cas9 had cleaved both gRNA recognition sites (the genome and the tia1l sequence) at the PAM −3 position and endogenous DNA ligases subsequently linked the two sequences without additional end processing. We noted that some clones show an identical mutation pattern (for example, 669-12 and 669-24). This indicates that there is either a dominant mutation pattern that could be favoured due to local sequence constraints or that these represent siblings from the same initial clone. Overall, the results indicate that this gene-tagging approach is remarkably precise and delivers clones in a predictable fashion.

### Generation of NanoLuc reporter cell lines

As we found that integration of the reporter cassette preferentially occurred exactly at the PAM −3 position (Fig. 2), we constructed three versions of a NanoLuc donor plasmid for each possible reading frame (Supplementary Fig. 3) to take into account where in the reading frame Cas9 introduces the cut. Furthermore, we neither included a start nor a stop codon in the donor to accommodate fusion seamlessly anywhere within the coding sequence of a gene (Supplementary Fig. 3).

**Figure 3:**
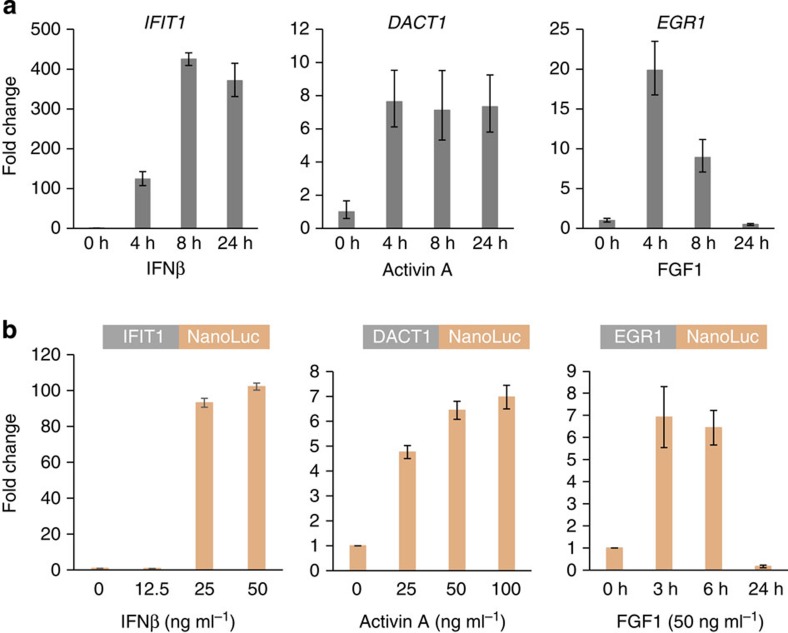
Cell lines bearing NanoLuc integrations can be used to monitor changes in gene expression. (**a**) HAP1 cells were stimulated with various cytokines as indicated (IFN-β, activin A and FGF1) for 4, 8 or 24 h at a final concentration of 50 ng ml^−1^. RNA was isolated and analysed by qPCR for the following signature genes: *IFIT1*, *DACT1* and EGR1. Error bars show the s.d. from three technical replicates. (**b**) Clonal cell lines bearing NanoLuc integrations in *IFIT1*, *DACT1* and *EGR1* (Table 2) were stimulated with IFN-β, activin A or FGF1 as indicated. Cell lines were collected after 24 h (for *IFIT1* and *DACT1*) or after the indicated time points (for *EGR1*) and NanoLuc luciferase levels were measured. Error bars show the s.d. from six technical replicates.

We now aimed to tag a set of genes with these altered NanoLuc reporter cassettes to allow for subsequent functional evaluation. NanoLuc^13^ was chosen, as it is small and bright and thus compatible with low signal intensities expected from a single genomic integration event. We decided to tag three cytokine-inducible genes (*DACT1*, *IFIT1* and *EGR1*)—*DACT1*/Dapper1 is a Wnt antagonist that is regulated by activin A^14^, *IFIT1* is an interferon-inducible gene^15^ and *EGR1* is regulated in response to various stimuli including FGF1 (ref. 16). We initially confirmed upregulation of the corresponding messenger RNAs in response to cytokine stimulation by quantitative PCR (qPCR) in wild-type HAP1 cells (Fig. 3a). Next we chose one gRNA for each gene and selected the appropriate NanoLuc cassette bearing the reading frame that would create an in-frame integration event if Cas9 cleavage and ligation occurred as predicted. Up to 96 clones were screened by PCR and Sanger sequencing across the 5′ junction. In line with our previous findings (Fig. 2; Table 1), two to seven clones were positive as judged by the PCR across the 5′ junction. The majority of these contained in-frame integrations (Table 2; Supplementary Fig. 4). However, not all of the clones bearing correct 5′ junctions also contained correct 3′ junctions (Table 2). Overall, we were able to identify one clone containing the perfect integration pattern for both *IFIT1* and *EGR1*. For *DACT1*, in contrast, all clones that bore the correct 5′ junction had imperfect 3′ junctions. However, since the tag was inserted at the 3′ end of *DACT1*, most of the *DACT1*-coding sequence was still unhampered. Thus, we decided to use one of the imperfect *DACT1*-NanoLuc clones for functional analysis.

Next, we tested the cell lines bearing defined NanoLuc integrations in *DACT1*, *IFIT1* and *EGR1* by stimulating them with their cognate cytokine ligands. All three tagged alleles showed cytokine-induced upregulation of NanoLuc levels as measured by the NanoGlo Luciferase assay system (Fig. 3b). Importantly, data obtained using NanoLuc measurements nicely reflected the qPCR data obtained previously (compare Fig. 3b with Fig. 3a), both with regard to the degree of cytokine stimulation and the kinetics. This suggests that NanoLuc reporter cell lines, engineered by our generic gene-tagging approach, can be used to faithfully monitor gene expression at the endogenous level.

### Generation of TurboGFP reporter cell lines

**Figure 4:**
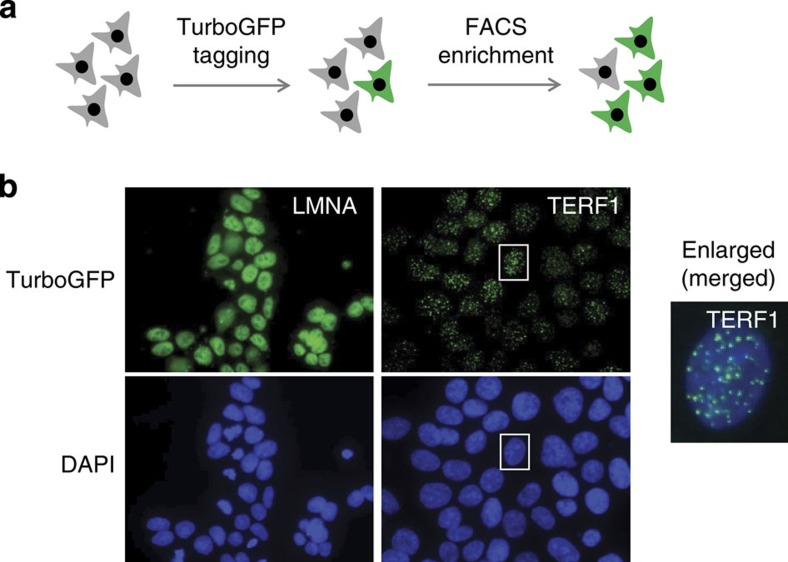
Cell lines bearing TurboGFP can be used to monitor subcellular localization. (**a**) HAP1 cells were transfected with expression plasmids for Cas9 and the generic TurboGFP donor that expresses the tia1l gRNA. In addition, we co-transfected one gRNA for each gene under consideration (*LMNA*, *TERF1* and *LAMP1*). TurboGFP-positive cells were enriched by FACS. (**b**) Clonal cell lines bearing TurboGFP integrations in *LMNA* and *TERF1* were fixed, stained with 4,6-diamidino-2-phenylindole (DAPI) and analysed by fluorescence microscopy.

We also assessed the feasibility of our approach for a different purpose: the tagging of endogenous genes with fluorescent markers suitable for live-cell imaging. We selected TurboGFP^17^ because it is very bright and photostable and allows the enrichment of cells bearing the tagged allele by fluorescence-activated cell sorting (FACS; Fig. 4a), which will increase the frequency by which genome-edited clones can be recovered. As outlined above for NanoLuc, we generated one cassette for each of the three reading frames to obtain three generic TurboGFP donors (Supplementary Fig. 5). We decided to tag three genes that display distinct subcellular localization patterns: *LMNA* encodes a component of the nuclear envelope (Lamin A)^18^. The *TERF1* gene product TRF1 binds to telomeres and is known to display a dotted nuclear pattern^19^. *LAMP1* encodes a lysosomal marker LAMP1 that is clustered in cytoplasmic aggregates^20^. For each of these genes, we selected a single gRNA targeting a region within the gene that is likely to tolerate a TurboGFP insertion (*LMNA* and *TERF1* at the 5′ end of the coding sequence/N- terminus of the protein, *LAMP1* at the 3′ end of the coding sequence/C terminus of the protein). HAP1 cells were then transfected with Cas9, a gRNA specifying the genomic locus and the appropriate TurboGFP donor plasmid. Following transfection, we observed that up to about 1% of the cells were positive for TurboGFP (Table 3). TurboGFP-positive cells were subsequently enriched by FACS and TurboGFP positivity increased roughly 50-fold in the sorted cell populations (Table 3). Single-cell clones were then isolated by limiting dilution. When analysing the 5′ junction for each of the genes, we observed high frequencies of cassette integration (2/19 for *LMNA*, 8/17 for *TERF1* and 9/19 for *LAMP1*; Table 4; Supplementary Fig. 6). The majority of the clones that displayed the correct 5′ junction also showed the correct 3′ junction (Table 4). This is in line with the notion that FACS clearly enriched for cells bearing correct tagging events.

Finally, we analysed correctly targeted TurboGFP clones by fluorescence microscopy. In all of the cases we assessed, the TurboGFP pattern was homogenous, as expected for a clonal cell line bearing a single genomic tagging event. Lamin A displayed nuclear staining in line with its localization at the nuclear membrane (Fig. 4b). In dividing cells that could be identified based on 4,6-diamidino-2-phenylindole staining, the distribution of Lamin A was more diffuse (Supplementary Fig. 7). This highlights the authenticity of the Lamin A localization pattern as Lamin A is relocalized during nuclear envelope breakdown. Localization of TRF1 was highly distinct with dotted clusters distributed over the entire nucleus (Fig. 4b). This is in agreement with TRF1 bound to telomeres. For LAMP1, the TurboGFP signal was more diffuse with occasional aggregates representing the formation of active lysosomes (Supplementary Fig. 8). In summary, these data suggest that cell lines bearing TurboGFP alleles can be used for imaging studies and that localization patterns observed accurately reflect localization of the endogenous gene products.

Finally, we wondered whether our approach would also be feasible in diploid human cells or whether it was confined to haploid cells that may harbour a different repertoire of DNA damage repair pathways. To this end, we transfected HEK293 cells with a gRNA targeting *LMNA* and the corresponding TurboGFP plasmid donor as described above and analysed the transfected pools by flow cytometry. Pools were positive for GFP and GFP positivity could be increased by FACS sorting (Supplementary Fig. 9A). Cassette integration could be detected by PCR in pools of FACS-sorted cells (Supplementary Fig. 9B). Single-cell clones bearing on-target integration events in *LMNA* were obtained at frequencies >50% (Supplementary Fig. 9C). Three cell lines derived from these single clones were confirmed to be GFP positive (Supplementary Fig. 9D). We also evaluated the copy number of TurboGFP in these three clonal cell lines (Supplementary Fig. 9E): two clones displayed a single integration event, while one clone showed a copy number of 2 for the TurboGFP cassette. These data suggest that biallelic targeting may be possible with our approach, while it does not rule out the possibility that the second TurboGFP integration event may have occurred at an off-target site. Overall, this set of experiments suggests that our gene-tagging approach is also feasible in other commonly used cell lines such as HEK293.

## Discussion

We present here a generic approach for the introduction of foreign sequences into the human genome. It is independent of homology templates, yet it works with surprising efficiency and precision. Using NanoLuc or TurboGFP, we demonstrate that tagged cell lines can be obtained and used to monitor endogenous gene expression (NanoLuc) or subcellular localization (TurboGFP). While this study was being revised, a similar approach was used to disrupt endogenous genes with a blasticidin marker^21^,^22^. This highlights the flexibility and the great potential of this approach.

In contrast to tagging by homology-directed repair, our approach is less precise as it depends on the availability of gRNAs in proximity to the site where the tag is to be introduced. This is a potential shortcoming with regard to proteins that may be sensitive to insertions at particular sites or that lack suitable gRNA recognition sites. However, gRNA target sites are quite frequent—given the PAM site requirement (NGG), they statistically occur every 16 bp—and thus, for any given gene, there is a good chance to identify a suitable gRNA cleavage site. Moreover, the availability of additional Cas9 variants, for example, Cas9 from *Staphylococcus aureus*^23^, will expand on the number of accessible sites in the human genome. Furthermore, our design of tagging cassettes without START or STOP codon allows for the integration of the tag anywhere within the gene; that is, both at the N or the C terminus or even at any other position with the reading frame, should that need arise.

In some instances, we were unable to identify clones bearing tagged alleles (Table 1). In these cases, we suspect that tagging is feasible in principle as evidenced by the integration of the cassette as detected by PCR in the pools of transfected cells (Fig. 1b), but most likely occurs at lower frequency and might thus not be identifiable by only screening a limited number of 24 single-cell clones. The frequency of donor integration may depend on several factors including gRNA cleavage efficiency^24^, local chromatin structure^24^ and microhomologies^25^ between the cassette and the genome. One potential solution for this problem is the use of selectable markers to increase chances of recovering cell lines bearing a tagged allele, as shown for TurboGFP-tagged alleles in Fig. 4a and Table 3.

Following FACS enrichment, we observed that a significant fraction of the clones did not contain TurboGFP integrations at the target site. The majority of these can be attributed to the fact that our FACS enrichment was incomplete, as a consequence of the low percentage of TurboGFP-positive cells in the initial population (Table 3). In addition, some of the clones may have escaped our analytical procedure due to a low cell number or suboptimal PCR conditions. Optimizing the conditions for FACS sorting and PCR analysis is likely to increase the overall yield of the procedure.

One additional concern of a homology-independent approach is off-target integration of the donor at an unintended site in the genome. This could either occur spontaneously or be triggered by Cas9/gRNA cleavage at an unwanted site. We noted that in some cases (for example, TurboGFP tagging of *LMNA*), off-target integration may have occurred at considerable frequencies as we only recovered few clones bearing an on-target integration from a pool of cells that was largely TurboGFP positive. The frequency of such undesired events will depend on the spontaneous integration rate of the donor, the cleavage efficiency at the on-target site and the specificity of the gRNA that was used.

We wondered whether the cell lines we generated contained a second integration event of the donor cassette. This is of particular concern with an approach that does not rely on homology-direct repair. To address this question, we subjected 15 cell lines bearing on-target integrations of NanoLuc or TurboGFP cassettes to droplet digital PCR (ddPCR) analysis^26^. Our analysis showed that most clones contained a single integration of the tagging cassette (Supplementary Fig. 10). The exception was clone *DACT1* E11 that contained a NanoLuc copy number of 0.5 compared with the RNAse P reference. This most likely represents a clone that turned diploid in the process and contained a single integration of the NanoLuc tag on one allele. We recommend ddPCR as a standard practice to exclude cell lines bearing more than one integration event.

All genes that we assessed in terms of gene tagging in this study are not essential in HAP1 cells according to a recent report^21^. When essential genes are being tagged, in-frame insertions may be favoured over out-of-frame integration events by natural selection, especially when tagging essential genes at the N terminus. Thus, the approach presented here should allow the tagging of essential genes if the tagged allele can maintain gene function.

While we believe that the approach presented here is very useful and broadly applicable, we admit that the underlying mechanism is poorly understood. Given the wealth of data showing the prevalence of indel formation after Cas9 cleavage, we were surprised to see that integration occurs in such a predictable and precise manner. On the one hand, this precision may reflect a favourable DNA damage repair configuration that is confined to certain species or cell types. On the other hand, the precision may arise from the use of incompatible gRNA recognition sites (genomic gRNA and tia1l gRNA). Following ligation, these sites are no longer accessible for Cas9 cleavage and hence, the accumulation of additional damaging mutations as a result of multiple cycles of Cas9 cleavage and NHEJ repair becomes less likely. When establishing the approach, we also tried using PCR products as donors, but this approach failed. We speculate that co-processing of the donor is required to deliver the donor to the right location at the right time. If a linear piece of DNA is co-transfected as a donor instead, we assume that this donor will either not get to the site of Cas9 cleavage or that it is degraded before it can be integrated.

In summary, the approach we have developed is robust, precise and scalable and opens up exciting opportunities, such as the establishment of a collection of cell lines bearing TurboGFP, NanoLuc or other tags.

## Methods

### Cell lines and growth conditions

HAP1 cells^11^,^12^ can be obtained from Horizon Genomics (Vienna, Austria). HAP1 cells were grown in Iscove's modified Dulbecco's medium (Gibco) supplemented with 10% fetal calf serum and 1% Pen-Strep (Gibco).

### Genome editing

We used an expression plasmid in which Cas9 from *Streptococcus pyogenes* was expressed from a CMV promoter^2^,^3^,^4^. gRNA sequences are shown in Tables 1,2 and 4. All donor cassettes are shown in Supplementary Figs 1,3 and 5.

Genome editing in HAP1 cells was performed as follows: in brief, we transfected HAP1 cells in six-well plates using Turbofectin (Origene). Cells were transfected with 1 μg of Cas9 expression vector, 800 ng of gRNA expression vector and 500 ng of donor vector (containing an expression cassette for the tia1l gRNA). To enrich for transfected cells, we co-transfected 200 ng of a plasmid encoding a blasticidin resistance gene and subjected cells to transient selection with 20 μg ml^−1^ blasticidin. Transfected cells were either expanded for genomic DNA isolation or for limiting dilution^12^.

### Genomic DNA isolation and PCR

Genomic DNA was isolated using the QIAamp DNA Mini Kit (Qiagen) according to manufacturer's instructions. PCR was performed using GoTaq Polymerase (Promega) according to manufacturer's instructions. To detect the integration event, we combined a primer binding in the cassette (NanoLuc or TurboGFP) with a gene-specific primer. For a list of all genotyping primers see Supplementary Table 1.

### T7 endonuclease assay

Cas9-mediated genome-editing efficiency for specific gRNAs was measured by T7 endonuclease assay as follows: in brief, PCR products were subjected to another round of denaturation and annealing, followed by digestion with the mismatch-sensitive T7 endonuclease I (NEB). Digested products were resolved on a 2% agarose gel^27^. Primers used for the amplification of the genomic region around the corresponding gRNA cleavage site are listed in Supplementary Table 2.

### Quantitative reverse transcription–PCR

RNA was isolated using the RNEasy Mini Kit (Qiagen) and converted to complementary DNA using reverse transcriptase (Fermentas) and oligo (dT) primer. Complementary DNA was analysed by qPCR using the KAPA SYBR FAST qPCR kit (Peqlab) and normalized to beta-actin. Primers used for qPCR analysis are listed in Supplementary Table 2.

### NanoLuc reporter assay

Cells were analysed using the NanoGlo Luciferase kit (Promega) according to manufacturer's instructions.

### TurboGFP microscopy

Cells were washed with PBS, fixed with 4% paraformaldehyde and permeabilized using 0.1% Triton X-100. Cells were stained with 50 ng ml^−1^ 4,6-diamidino-2-phenylindole in PBS, washed with PBS and mounted in Prolong Gold Antifade Mountant (Life Technologies).

### Droplet digital PCR

Genomic DNA was analysed by ddPCR (BioRad). All primer and probe sequences are listed in Supplementary Table 2. RNAse P was used a reference to determine the absolute copy number for NanoLuc or TurboGFP. Samples were analysed using the QX100 droplet reader (BioRad). We included genomic DNA from parental HAP1 cells as negative control.

## 

## Additional information

**How to cite this article**: Lackner, D.H. *et al.* A generic strategy for CRISPR-Cas9-mediated gene tagging. *Nat. Commun.* 6:10237 doi: 10.1038/ncomms10237 (2015).

## Supplementary Material

Supplementary Information Supplementary Figures 1-10 and Supplementary Tables 1-2

## Figures and Tables

**Table 1:** Identification of single-cell clones bearing NanoLuc integrations.

**Gene**	**gRNA ID**	**gRNA sequence**	**Tagged clones/total clones**
ID1	2655	5′-GTGCTGAGCGGAGCCCGGAC-3′	2/24
ID1	2656	5′-GGCGCTGATCTCGCCGTTGA-3′	5/24
IRF9	2659	5′-GGCCTTTGCCCGATACTTGC-3′	1/24
IRF9	2660	5′-AGTCTGCTCCAGCAAGTATC-3′	0/24
TAP2	2663	5′-TGCGGGACAGAAACAACGTC-3′	0/24
TAP2	2664	5′-CATCCAGGATGAGGACCCGC-3′	0/24
CCL2	2665	5′-ACAGATCTCCTTGGCCACAA-3′	0/24
CCL2	2666	5′-TCAGGATTCCATGGACCACC-3′	1/24
IL6	669	5′-GTGCCTGCAGCTTCGTCAGC-3′	3/24

gRNA, guide RNA.

Pools of transfected cells described in Fig. 1 were subjected to limiting dilution and 24 single-cell clones were isolated per condition. Each clone was genotyped by PCR as described in Fig. 1 to identify clones bearing NanoLuc integrations. Identity of PCR products was confirmed by Sanger sequencing.

**Table 2:** Identification of single-cell clones bearing NanoLuc integrations in correct reading frame.

		**PCR positive**		**In-frame integration**	
**Gene**	**gRNA sequence**	**5′ junction**	**Both junctions**	**5′ junction**	**Both junctions**
IFIT1	5′-CCAATTTGTAGACGAACCCA-3′	7/84	4/7	2/4	1/2
DACT1	5′-ACAAGCGAACTGACTACCGG-3′	5/96	3/5	3/3	0/3
EGR1	5′-GTACGTGGTGGCCACCGACG-3′	2/96	1/2	1/1	1/1

gRNA, guide RNA.

HAP1 cells were transfected with expression plasmids for Cas9, the generic NanoLuc donor specified in Supplementary Fig. 3 and one gRNA for each gene under consideration (*IFIT1*, *DACT1*, *EGR1*). Single-cell clones were isolated by limiting dilution and analysed by PCR and Sanger sequencing.

**Table 3:** Cells bearing TurboGFP-tagged alleles can be enriched by FACS sorting.

**Gene**	**GFP positive (pre-sorting)**	**GFP positive (post-sorting)**
LMNA	1.1%	75.9%
TERF1	0.4%	21.5%
LAMP1	0.4%	26.3%

FACS, fluorescence-activated cell sorting; GFP, green fluorescent protein; gRNA, guide RNA.

HAP1 cells containing TurboGFP-tagged LMNA, TERF1 or LAMP1 were analysed for GFP positivity by FACS, either before or after FACS sorting.

**Table 4:** Identification of single-cell clones bearing in-frame integrations of TurboGFP.

		**PCR positive**		**In-frame integration**	
**Gene**	**gRNA sequence**	**5′ junction**	**Both junctions**	**5′ junction**	**Both junctions**
LMNA	5′-GGAGCTCAATGATCGCTTGG-3′	2/19	2/2	2/2	2/2
TERF1	5′-TACCATCCGCACAGCCCCGC-3′	8/17	8/8	3/8	3/3
LAMP1	5′-TCATCGCCTACCTCGTCGGC-3′	9/19	9/9	4/9	3/4

gRNA, guide RNA.

Analysis of single-cell clones derived from limiting dilutions carrying TurboGFP integrations (as described in Fig. 4). Clones were analysed by PCR and Sanger sequencing.
