# Supplementary material for: A generic strategy for CRISPR-Cas9-mediated gene tagging
Source: Nat Commun. 2015 Dec 17;6:10237. doi: 10.1038/ncomms10237 (PMC4703899; doi:10.1038/ncomms10237)
Supplement: Supplementary Information — Supplementary Figures 1-10 and Supplementary Tables 1-2 [file ncomms10237-s1.pdf]

Tia1l guide RNA sequence from 5’ to 3’ GGTATGTCGGGAACCTCTCC

PAM associated with the tia1l guide RNA from 5’ to 3’ AGG

NanoLuc cassette with β-globin polyadenylation signal

GAATTCGGTATGTCGGGAACCTCTCCAGGGGCAGCGGATCCATGGTCTTCACACTCGAAGATTTCGTT
GGGGACTGGCGACAGACAGCCGGCTACAACCTGGACCAAGTCCTTGAACAGGGAGGTGTGTCCAGTTT
GTTTCAGAATCTCGGGGTGTCCGTAACTCCGATCCAAAGGATTGTCCTGAGCGGTGAAAATGGGCTGA
AGATCGACATCCATGTCATCATCCCGTATGAAGGTCTGAGCGGCGACCAAATGGGCCAGATCGAAAAA
ATTTTTAAGGTGGTGTACCCTGTGGATGATCATCACTTTAAGGTGATCCTGCACTATGGCACACTGGT
AATCGACGGGGTTACGCCGAACATGATCGACTATTTCGGACGGCCGTATGAAGGCATCGCCGTGTTCG
ACGGCAAAAAGATCACTGTAACAGGGACCCTGTGGAACGGCAACAAAATTATCGACGAGCGCCTGATC
AACCCCGACGGCTCCCTGCTGTTCCGAGTAACCATCAACGGAGTGACCGGCTGGCGGCTGTGCGAACG
CATTCTGGCGTAATTCTAGAAGCTCGCTTTCTTGCTGTCCAATTTCTATTAAAGGTTCCTTTGTTCCC
TAAGTCCAACTACTAAACTGGGGGATATTATGAAGGGCCTTGAGCATCTGGATTCTGCCTAATAAAAA
ACATTTATTTTCATTGCAATGATGTATTTAAATTATTTCTGAATATTTTACTAAAAAGGGAATGTGGG
AGGTCAGTGCATTTAAAACATAAAGAAATGAAGAGCTAGTTCAAACCTTGGGAAAATACACTATATCT
TAAACTCCATGAAAGAAGGTGAGGCTGCAAACAGCTAATGCACATTGGCAACAGCCCCTGATGCCTAT
GCCTTATTCATCCCTCAGAAAAGGATTCAAGTAGAGGCTTGATTTGGAGGTTAAAGTTTTGCTATGCT
GTATTTTAGGATCCCACCGGGTCTTCGAGAAGACCCTGGAGAGGTTCCCGACATACCAGCTGCAG

Supplementary Figure 1: Architecture of first-generation NanoLuc donor cassette. For the experiments presented in Fig. 1 and 2, we used a donor containing the ORF for NanoLuc (orange), followed by the β-globin polyadenylation signal (blue). Tia1l gRNA sites and PAM site are marked in green and yellow, respectively.

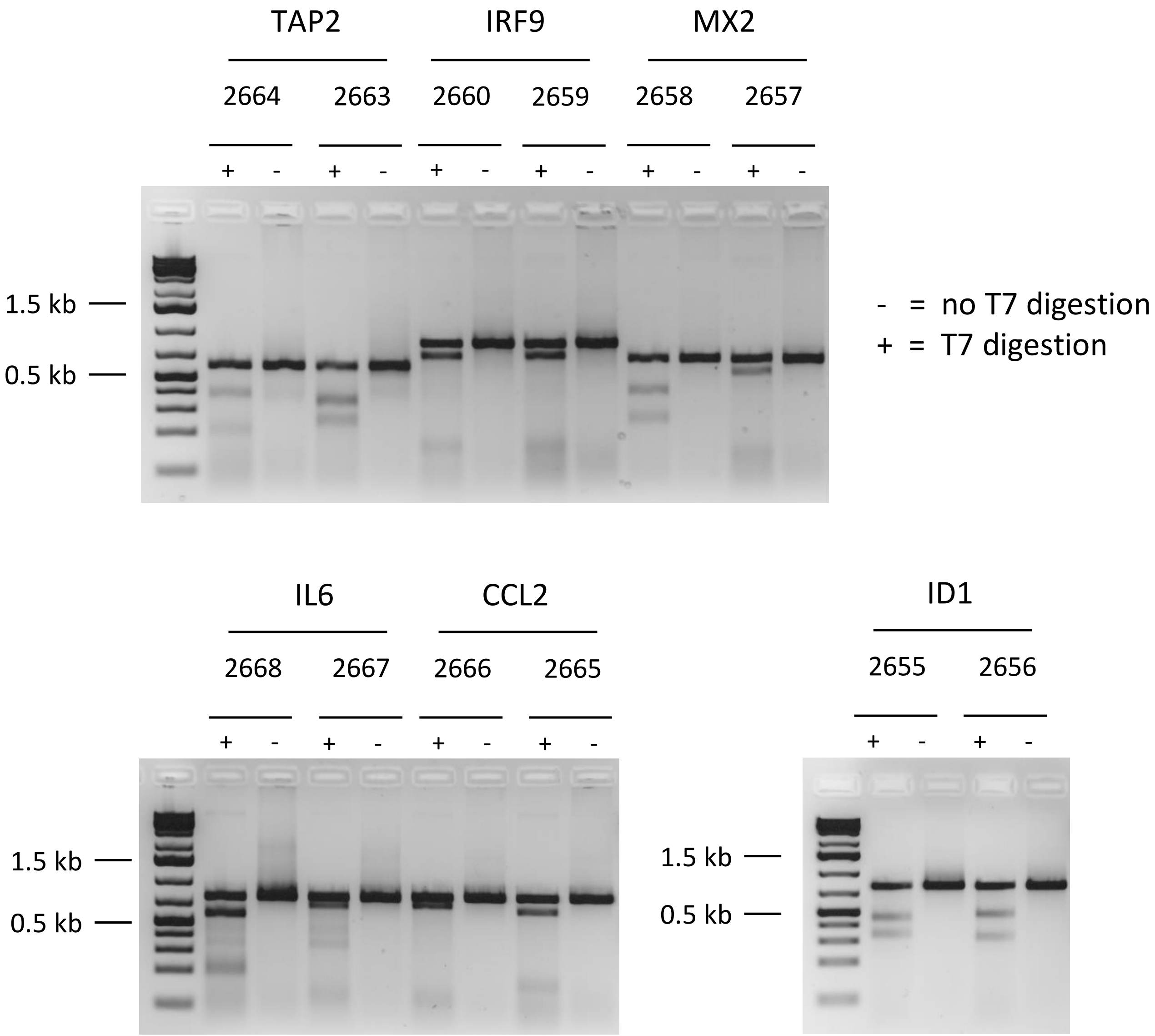

Supplementary Figure 2: T7 endonuclease assays. T7 endonuclease assays to test cleavage efficiency of gRNAs used in Fig. 1 and Fig. 2. PCRs spanning the gRNA sites of the indicated genes were performed on gDNA samples from HAP1 cells transfected with the indicated gRNAs and Cas9. PCR products were loaded directly on an agarose gel or after digestion with T7 endonuclease. Numbers above the lanes indicate the respective gRNAs as specified in Fig. 1B and Fig. 2.

Tia1l guide RNA sequence from 5’ to 3’	GGTATGTCGGGAACCTCTCC
PAM associated with the tia1l guide RNA from 5’ to 3’	AGG
Additional base pairs	G

NanoLuc cassette +0

GAATTCGGTATGTCGGGAACCTCTCCAGGGGATCCGTCTTCACACTCGAAGATTTCGTTGGGGACTGG
CGACAGACAGCCGGCTACAACCTGGACCAAGTCCTTGAACAGGGAGGTGTGTCCAGTTTGTTTCAGAA
TCTCGGGGTGTCCGTAACTCCGATCCAAAGGATTGTCCTGAGCGGTGAAAATGGGCTGAAGATCGACA
TCCATGTCATCATCCCGTATGAAGGTCTGAGCGGCGACCAAATGGGCCAGATCGAAAAAATTTTTAAG
GTGGTGTACCCTGTGGATGATCATCACTTTAAGGTGATCCTGCACTATGGCACACTGGTAATCGACGG
GGTTACGCCGAACATGATCGACTATTTCGGACGGCCGTATGAAGGCATCGCCGTGTTCGACGGCAAAA
AGATCACTGTAACAGGGACCCTGTGGAACGGCAACAAAATTATCGACGAGCGCCTGATCAACCCCGAC
GGCTCCCTGCTGTTCCGAGTAACCATCAACGGAGTGACCGGCTGGCGGCTGTGCGAACGCATTCTGGC
GGGATCCCCTGGAGAGGTTCCCGACATACCCACTG

NanoLuc cassette +1

GAATTCGGTATGTCGGGAACCTCTCCAGGGGGATCCGTCTTCACACTCGAAGATTTCGTTGGGGACTG
GCGACAGACAGCCGGCTACAACCTGGACCAAGTCCTTGAACAGGGAGGTGTGTCCAGTTTGTTTCAGA
ATCTCGGGGTGTCCGTAACTCCGATCCAAAGGATTGTCCTGAGCGGTGAAAATGGGCTGAAGATCGAC
ATCCATGTCATCATCCCGTATGAAGGTCTGAGCGGCGACCAAATGGGCCAGATCGAAAAAATTTTTAA
GGTGGTGTACCCTGTGGATGATCATCACTTTAAGGTGATCCTGCACTATGGCACACTGGTAATCGACG
GGGTTACGCCGAACATGATCGACTATTTCGGACGGCCGTATGAAGGCATCGCCGTGTTCGACGGCAAA
AAGATCACTGTAACAGGGACCCTGTGGAACGGCAACAAAATTATCGACGAGCGCCTGATCAACCCCGA
CGGCTCCCTGCTGTTCCGAGTAACCATCAACGGAGTGACCGGCTGGCGGCTGTGCGAACGCATTCTGG
CGGGATCCGACCTGGAGAGGTTCCCGACATACCCACTG

NanoLuc cassette +2

GAATTCGGTATGTCGGGAACCTCTCCAGGGAGGATCCGTCTTCACACTCGAAGATTTCGTTGGGGACT
GGCGACAGACAGCCGGCTACAACCTGGACCAAGTCCTTGAACAGGGAGGTGTGTCCAGTTTGTTTCAG
AATCTCGGGGTGTCCGTAACTCCGATCCAAAGGATTGTCCTGAGCGGTGAAAATGGGCTGAAGATCGA
CATCCATGTCATCATCCCGTATGAAGGTCTGAGCGGCGACCAAATGGGCCAGATCGAAAAAATTTTTA
AGGTGGTGTACCCTGTGGATGATCATCACTTTAAGGTGATCCTGCACTATGGCACACTGGTAATCGAC
GGGGTTACGCCGAACATGATCGACTATTTCGGACGGCCGTATGAAGGCATCGCCGTGTTCGACGGCAA
AAAGATCACTGTAACAGGGACCCTGTGGAACGGCAACAAAATTATCGACGAGCGCCTGATCAACCCCG
ACGGCTCCCTGCTGTTCCGAGTAACCATCAACGGAGTGACCGGCTGGCGGCTGTGCGAACGCATTCTG
GCGGGATCCACCTGGAGAGGTTCCCGACATACCCACTG

Supplementary Figure 3: Architecture of second-generation NanoLuc reporter cassettes. For the experiments presented in Fig. 3, we used a set of donors containing the ORF for NanoLuc (orange) in the three possible reading frames. Additional bases that are needed to maintain the reading frame are colored in red. Tia1l gRNA sites and PAM site are marked in green and yellow, respectively.

DACT1-NanoLuc, clone A6 and G5 (NanoLuc cassette +0)

5' junction

```
5' Genome                          tia1l        NanoLuc                3'
GCCAAACCTAAGCACAAGCGAACTGACTACTCCAGGGGATCCGTCTTCACACTCGAAGAT
 A  K  P  K  H  K  R  T  D  Y  S  R  G  S  V  F  T  L  E  D
```

3' junction

```
5' NanoLuc                   tia1l          Genome                     3'
TGCGAACGCATTCTGGCGGGATCCCCTGGACCGGCGGTGGAAGTCCTCGGCCGAGATTTC
 C  E  R  I  L  A  G  S  P  G  P  A  V  E  V  L  G  R  D  F
```

IFIT1-NanoLuc, clone A9 (NanoLuc cassette +1)

5' junction

```
5' Genome                        tia1l          NanoLuc                3'
TTAGATCTGGAAAGCTTGAGCCTCCTTGGTCCAGGGGGATCCGTCTTCACACTCGAA
 L  D  L  E  S  L  S  L  L  G  P  G  G  S  V  F  T  L  E
```

3' junction

```
5' NanoLuc                       tia1l         Genome                  3'
TGCGAACGCATTCTGGCGGGATCCGACCTGGAGTTCGTCTACAAATTGGAAGGAAATATG
 C  E  R  I  L  A  G  S  D  L  E  F  V  Y  K  L  E  G  N  M
```

EGR1-NanoLuc, clone D6 (NanoLuc cassette +2)

5' junction

```
5' Genome                        tia1l            NanoLuc              3'
CCTGTGCACAGTGGCTTCCCCTCCCCGTTCCAGGGAGGATCCGTCTTCACACTCGAAGAT
 P  V  H  S  G  F  P  S  P  F  Q  G  G  S  V  F  T  L  E  D
```

3' junction

```
5' NanoLuc                    tia1l          Genome                    3'
TGCGAACGCATTCTGGCGGGATCCACCTGGACGGTGGCCACCACGTACTCCTCTGTTCCC
 C  E  R  I  L  A  G  S  T  W  T  V  A  T  T  Y  S  S  V  P
```

Supplementary Figure 4: Sequencing results for NanoLuc-tagged clones. HAP1 clones bearing NanoLuc integration events in DACT1, IFIT1 or EGR1 were characterized by PCR and Sanger sequencing across both junctions (5' and 3' junction). NanoLuc sequences are highlighted in orange. Tia1l gRNA sites and PAM site are marked in green and yellow, respectively.

Tia1l guide RNA sequence from 5’ to 3’ GGTATGTCGGGAACCTCTCC

PAM associated with the tia1l guide RNA from 5’ to 3’ AGG

Additional base pairs G

TurboGFP cassette+0

GAATTCGGTATGTCGGGAACCTCTCCAGGGGATCCGAGAGCGACGAGAGCGGCCTGCCCGCCATGGAGATCGAGT
GCCGCATCACCGGCACCCTGAACGGCGTGGAGTTCGAGCTGGTGGGCGGCGGAGAGGGCACCCCCGAGCAGGGCC
GCATGACCAACAAGATGAAGAGCACCAAAGGCGCCCTGACCTTCAGCCCCTACCTGCTGAGCCACGTGATGGGCT
ACGGCTTCTACCACTTCGGCACCTACCCCAGCGGCTACGAGAACCCCTTCCTGCACGCCATCAACAACGGCGGCT
ACACCAACACCCGCATCGAGAAGTACGAGGACGGCGGCGTGCTGCACGTGAGCTTCAGCTACCGCTACGAGGCCG
GCCGCGTGATCGGCGACTTCAAGGTGATGGGCACCGGCTTCCCCGAGGACAGCGTGATCTTCACCGACAAGATCA
TCCGCAGCAACGCCACCGTGGAGCACCTGCACCCCATGGGCGATAACGATCTGGATGGCAGCTTCACCCGCACCT
TCAGCCTGCGCGACGGCGGCTACTACAGCTCCGTGGTGGACAGCCACATGCACTTCAAGAGCGCCATCCACCCCA
GCATCCTGCAGAACGGGGGCCCCATGTTCGCCTTCCGCCGCGTGGAGGAGGATCACAGCAACACCGAGCTGGGCA
TCGTGGAGTACCAGCACGCCTTCAAGACCCCGGATGCAGATGCCGGTGAAGAAGGATCCCCTGGAGAGGTTCCCG
ACATACCCACTGCAG

TurboGFP cassette +1

GAATTCGGTATGTCGGGAACCTCTCCAGGGGGATCCGAGAGCGACGAGAGCGGCCTGCCCGCCATGGAGATCGAG
TGCCGCATCACCGGCACCCTGAACGGCGTGGAGTTCGAGCTGGTGGGCGGCGGAGAGGGCACCCCCGAGCAGGGC
CGCATGACCAACAAGATGAAGAGCACCAAAGGCGCCCTGACCTTCAGCCCCTACCTGCTGAGCCACGTGATGGGC
TACGGCTTCTACCACTTCGGCACCTACCCCAGCGGCTACGAGAACCCCTTCCTGCACGCCATCAACAACGGCGGC
TACACCAACACCCGCATCGAGAAGTACGAGGACGGCGGCGTGCTGCACGTGAGCTTCAGCTACCGCTACGAGGCC
GGCCGCGTGATCGGCGACTTCAAGGTGATGGGCACCGGCTTCCCCGAGGACAGCGTGATCTTCACCGACAAGATC
ATCCGCAGCAACGCCACCGTGGAGCACCTGCACCCCATGGGCGATAACGATCTGGATGGCAGCTTCACCCGCACC
TTCAGCCTGCGCGACGGCGGCTACTACAGCTCCGTGGTGGACAGCCACATGCACTTCAAGAGCGCCATCCACCCC
AGCATCCTGCAGAACGGGGGCCCCATGTTCGCCTTCCGCCGCGTGGAGGAGGATCACAGCAACACCGAGCTGGGC
ATCGTGGAGTACCAGCACGCCTTCAAGACCCCGGATGCAGATGCCGGTGAAGAAGGATCCGACCTGGAGAGGTTC
CCGACATACCCACTGCAG

TurboGFP cassette +2

GAATTCGGTATGTCGGGAACCTCTCCAGGGAGGATCCGAGAGCGACGAGAGCGGCCTGCCCGCCATGGAGATCGA
GTGCCGCATCACCGGCACCCTGAACGGCGTGGAGTTCGAGCTGGTGGGCGGCGGAGAGGGCACCCCCGAGCAGGG
CCGCATGACCAACAAGATGAAGAGCACCAAAGGCGCCCTGACCTTCAGCCCCTACCTGCTGAGCCACGTGATGGG
CTACGGCTTCTACCACTTCGGCACCTACCCCAGCGGCTACGAGAACCCCTTCCTGCACGCCATCAACAACGGCGG
CTACACCAACACCCGCATCGAGAAGTACGAGGACGGCGGCGTGCTGCACGTGAGCTTCAGCTACCGCTACGAGGC
CGGCCGCGTGATCGGCGACTTCAAGGTGATGGGCACCGGCTTCCCCGAGGACAGCGTGATCTTCACCGACAAGAT
CATCCGCAGCAACGCCACCGTGGAGCACCTGCACCCCATGGGCGATAACGATCTGGATGGCAGCTTCACCCGCAC
CTTCAGCCTGCGCGACGGCGGCTACTACAGCTCCGTGGTGGACAGCCACATGCACTTCAAGAGCGCCATCCACCC
CAGCATCCTGCAGAACGGGGGCCCCATGTTCGCCTTCCGCCGCGTGGAGGAGGATCACAGCAACACCGAGCTGGG
CATCGTGGAGTACCAGCACGCCTTCAAGACCCCGGATGCAGATGCCGGTGAAGAAGGATCCACCTGGAGAGGTTC
CCGACATACCCACTGCAG

Supplementary Figure 5: Architecture of TurboGFP reporter cassettes. For the experiments presented in Fig. 4, we used a set of donors containing the ORF for TurboGFP (dark green) in the three possible reading frames. Additional bases that are needed to maintain the reading frame are colored in red. Tia1l gRNA sites and PAM site are marked in green and yellow, respectively.

LAMP1-TurboGFP, clones A4, B12 and C2 (TurboGFP cassette +0)

5' junction

```
5' Genome                            tia1l          TurboGFP          3'

GTCCTCATCGTCCTCATCGCCTACCTCGTCTCCAGGGGATCCGAGAGCGACGAGAGCGGC
 V  L  I  V  L  I  A  Y  L  V  S  R  G  S  E  S  D  E  S  G
```

3' junction

```
5' TurboGFP                    tia1l            Genome                3'

GCAGATGCCGGTGAAGAAGGATCCCCTGGAGGCAGGAAGAGGAGTCACGCAGGCTACCAG
 A  D  A  G  E  E  G  S  P  G  G  R  K  R  S  H  A  G  Y  Q
```

TurboGFP-TERF1, clones A9, C3 and C5 (TurboGFP cassette +1)

5' junction

```
5' Genome                            tia1l        TurboGFP            3'

GATGTTTCCTCAGCGGCCCCGAGCCCGCGTCCAGGGGGATCCGAGAGCGACGAGAGCGGC
 D  V  S  S  A  A  P  S  P  R  P  G  G  S  E  S  D  E  S  G
```

3' junction

```
5' TurboGFP                       tia1l        Genome                 3'

GCAGATGCCGGTGAAGAAGGATCCGACCTGGAGGGCTGTGCGGATGGTAGGGATGCCGAC
 A  D  A  G  E  E  G  S  D  L  E  G  C  A  D  G  R  D  A  D
```

TurboGFP-LMNA, clones A3 and B9 (TurboGFP cassette +2)

5' junction

```
5' Genome                           tia1l          TurboGFP           3'

GAGGACCTGCAGGAGCTCAATGATCGCTTCCAGGGAGGATCCGAGAGCGACGAGAGCGGC
 E  D  L  Q  E  L  N  D  R  F  Q  G  G  S  E  S  D  E  S  G
```

3' junction

```
5' TurboGFP                     tia1l            Genome               3'

GCAGATGCCGGTGAAGAAGGATCCACCTGGATGGCGGTCTACATCGACCGTGTGCGCTCG
 A  D  A  G  E  E  G  S  T  W  M  A  V  Y  I  D  R  V  R  S
```

Supplementary Figure 6: Sequencing results for TurboGFP-tagged clones. HAP1 clones bearing TurboGFP integration events in LMNA, TERF1 or LAMP1 were characterized by PCR and Sanger sequencing across both junctions (5’ and 3’ junction). TurboGFP sequences are highlighted in dark green. Tia1l gRNA sites and PAM site are marked in green and yellow, respectively.

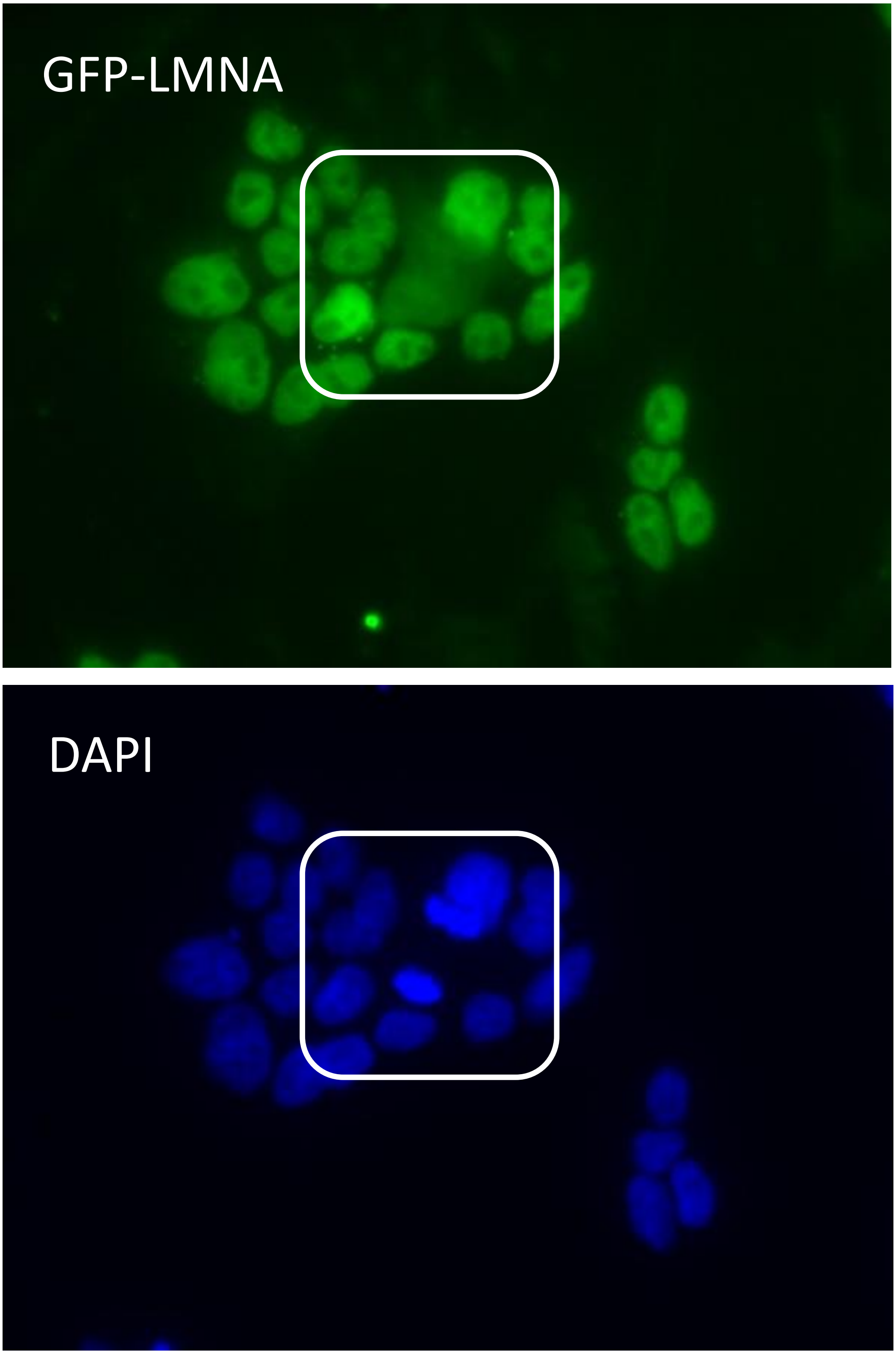

Supplementary Figure 7: Microscopy image of TurboGFP-tagged LMNA. Fluorescence image showing TurboGFP-tagged LMNA cells, counter-stained with DAPI. The white square indicates a cell undergoing mitosis.

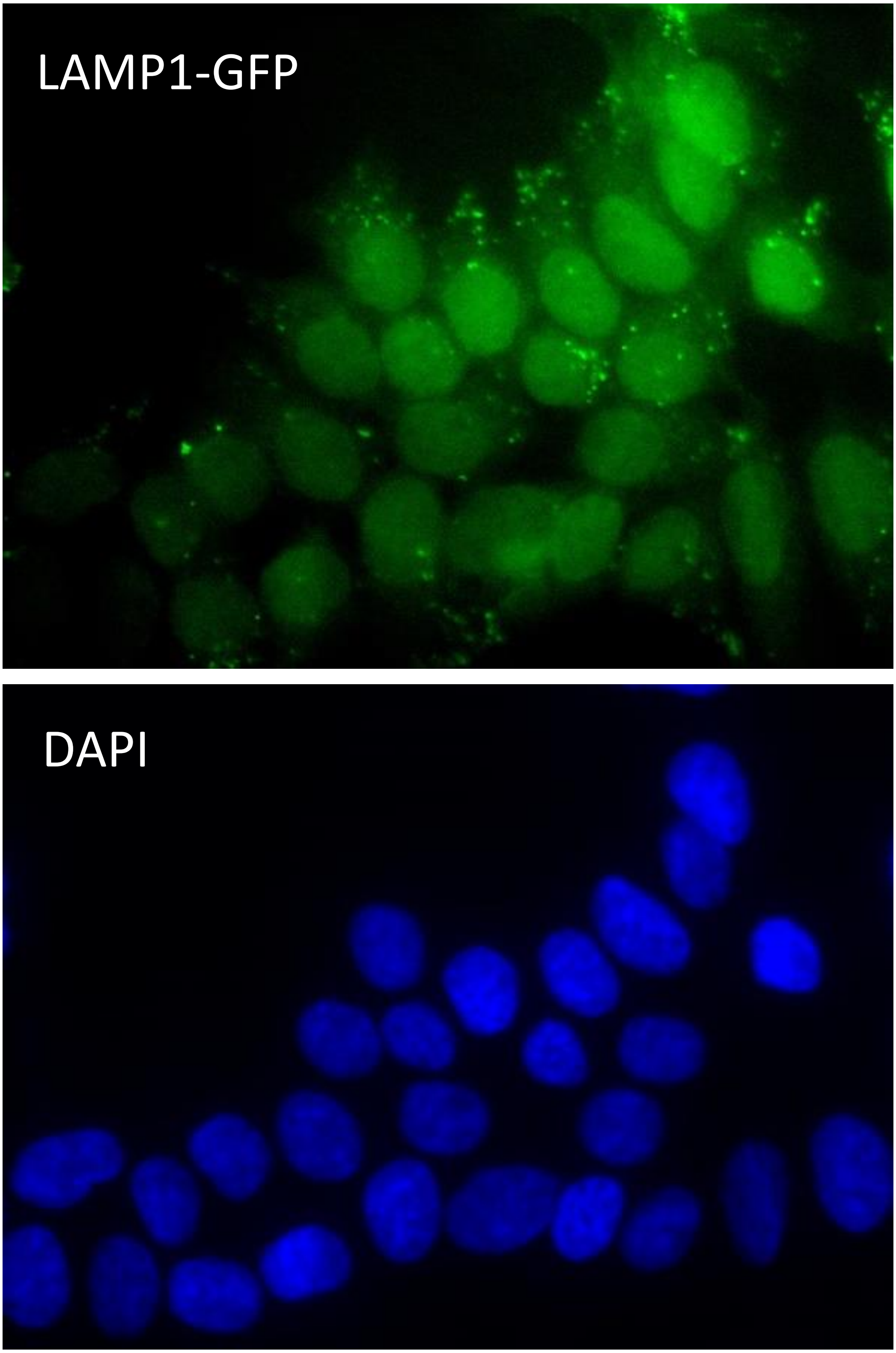

Supplementary Figure 8: Microscopy image of TurboGFP-tagged LAMP1. Fluorescence image showing TurboGFP-tagged LAMP1 cells, counter-stained with DAPI.

(A)

Gene	GFP-positive (pre-sorting)	GFP-positive (post-sorting)
LMNA	1.0%	89.5%

(B)

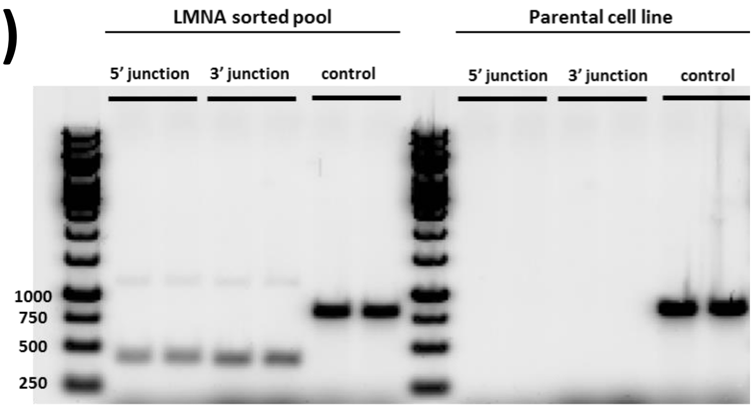

(C)

	PCR positive			In-frame integration	
Gene	# colonies screened by PCR	5ʻ junction	Both junctions	# clones sequenced	Both junctions
LMNA	470	341/470	270/341	12	12/12

(D)

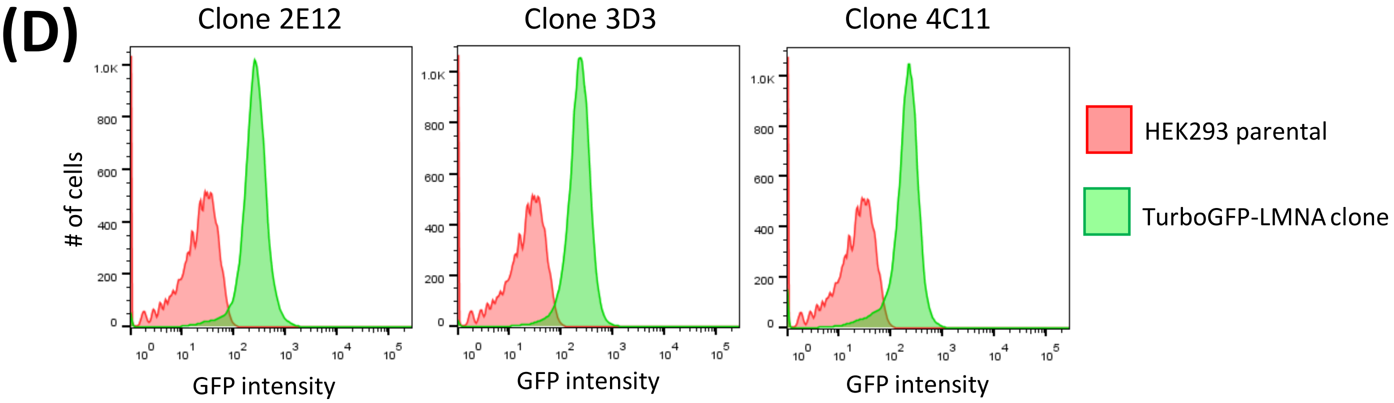

(E)

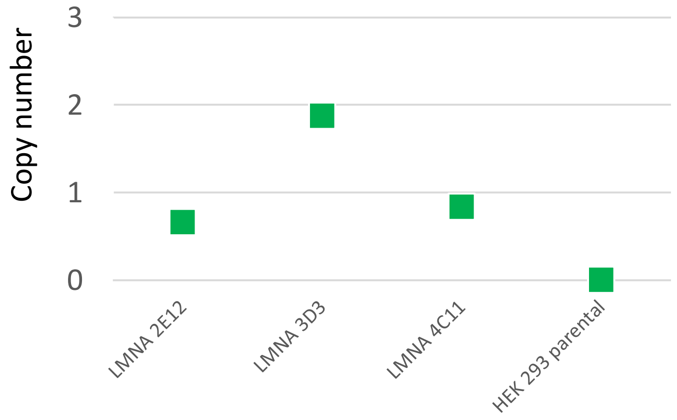

Supplementary Figure 9: TurboGFP tagging in diploid HEK293 cells. (A) GFP positivity as assessed by flow cytometry in cells after transfection and after enrichment by FACS is shown. (B) PCR products showing integration of the TurboGFP-tagging cassette in LMNA similarly as shown in Fig. 1B. For this PCR, one constant primer binding to the TurboGFP cassette was combined with one variable primer in the LMNA genomic locus to test both the 5' and 3' junction of the integrated cassette. Each PCR was run in duplicate. Positive control PCRs show a reference PCR on genomic DNA in the POLQ locus (FW primer: CCTTCCCCACGAGTCTATGG, REV primer: GTTCCATGACGACTGCACAA). (C) Analysis of single clones by PCR and Sanger sequencing. (D) Flow cytometry analysis of 3 TurboGFP-tagged LMNA clones. The red graph indicates GFP intensity in the parental HEK293 cell line, the green graph indicates GFP intensity in the corresponding TurboGFP-LMNA clone. (E) Droplet digital PCR (ddPCR) was used to assess copy number of the TurboGFP cassette in the indicate clones.

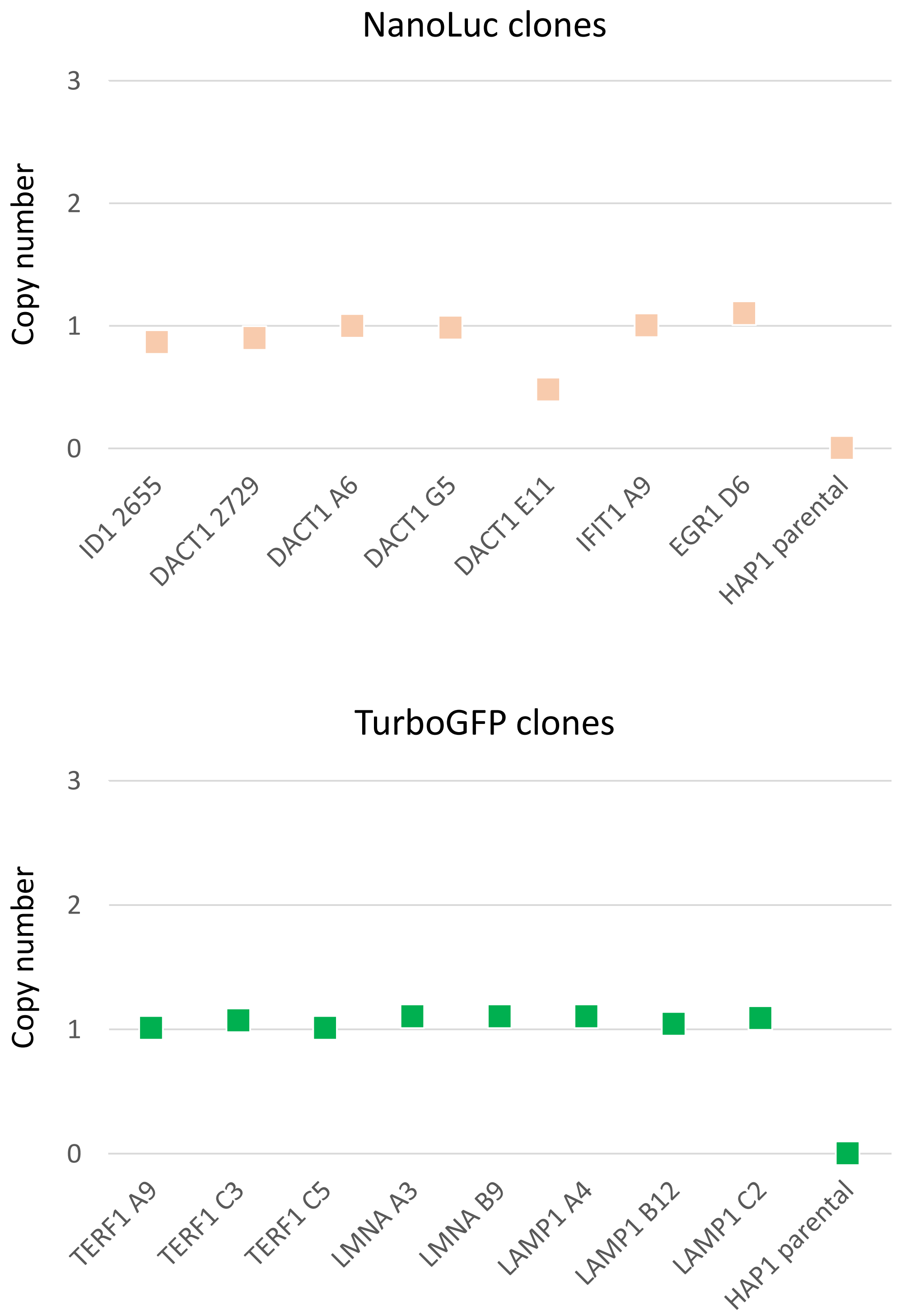

Supplementary Figure 10: Droplet Digital PCR (ddPCR) from HAP1 clones bearing cassette integrations. 7 HAP1 clones bearing NanoLuc integrations and 8 HAP1 clones bearing TurboGFP integrations were assed for copy number of the tagging cassette by ddPCR. HAP1 parental cells were included as reference.

Supplementary Table 1

Primers used for genotyping

Cassette-specific primers		
NanoLuc 5’ end	GGATCGGAGTTACGGACACC	
NanoLuc 3’ end	TCCGAGTAACCATCAACGGA	
TurboGFP 5’ end	CCGAAGTGGTAGAAGCCGTAG	
TurboGFP 3’ end	TGGGCGATAACGATCTGGATG	
**Gene-specific primers**		
**Gene**	**5‘ junction**	**3‘junction**
ID1	CTACGACATGAACGGCTGTTACTC	n/a
MX2	CTAGAGGGGATGCCTGGAAGTAAAG	n/a
IRF9	GAGTACCCATCTAATGAGAGCAGAG	n/a
TAP2	GAAGTACCTGCTGTGCACTTGTC	n/a
CCL2	GAACACCCCAATTTCTTTAGCTTGA	n/a
IL9	CTCATTCACCCCACATTTCACATTT	n/a
IFIT1	GTGGTAGAAGAAACAATGCAAGACA	TGAAATGAAATGTGAAAGTGGCTGA
DACT1	TAAGAAACTCAAGAAAGCCTCCTCC	CAGTTGGTGGTGTAATTGCTCTG
EGR1	AGAAGGACAAGAAAGCAGACAAAAG	TAGCAAATTTCAATTGTCCTGGGAG
LMNA	GAGCAGTCTCTGTCCTTCGAC	CTTGGCTACTGAGTCAAGGGTCTT
TERF1	TTGAACCGCTCGCCCATGT	AAAGAGAGAGGCAGAGGAAATCG
LAMP1	CTCTTTGCCTTTTGGCTGATGTG	ATGAACTTCACATTGGATGAAGCAG

Supplementary Table 2

Primers used for T7 endonuclease assays

ID1 FW	TATCTGCTTCGGGCTTCCAC
ID1 REV	TCAGCGACACAAGATGCGA
MX2 FW	TGGTGCATACCCAGAAGGGA
MX2 REV	CTGCACCGACTCCCCTTAGA
IRF9 FW	CAGAGTGTGGGTGTTCCCAG
IRF9 REV	AGAGGACAGGTCAATCGTGTG
TAP2 FW	GTTCCTCTGCCCTTTCCTCC
TAP2 REV	ACCGGATTCCATTCCCCAAC
CCL2 FW	CCTGCCTGCCTTTTGCTTTT
CCL2 REV	GTGTCTGGGGAAAGCTAGGG
IL6 FW	TGCTTGTGGTCTAATGGGAGAC
IL6 REV	CAACATAAGTTCTGTGCCCAGTG

Primers used for qPCR analysis

DACT1_qPCR_FW	GGGCTTCAGGATCCCTTTCC
DACT1_qPCR_REV	TCTGCAGATTTGGGGCAAC
EGR1_qPCR_FW	AGTCCCATTTACTCAGCGGC
EGR1_qPCR_REV	GTGGAAACAGGTAGTCGGGG
IFIT1_qPCR_FW	GTGCTTGAAGTGGACCCTGA
IFIT1_qPCR_REV	CCTGCCTTAGGGGAAGCAAA
ACTB_qPCR_FW	GTACCACTGGCATCGTGATGGACT
ACTB_qPCR_REV	CCGCTCATTGCCAATGGTGAT

Primers and probes used for ddPCR analysis

FWD primer NanoLuc	ACCAAATGGGCCAGATCGAAAA
REV primer NanoLuc	CCATAGTGCAGGATCACCTTAAAGT
Probe NanoLuc	FAM-ATGATCATCCACAGGGTACACCAC-MGB
FWD primer TurboGFP	CTGCACGTGAGCTTCAGCTA
REV primer TurboGFP	AAGCCGGTGCCCATCA
Probe TurboGFP	FAM-CCGCGTGATCGGCGACTT-MGB
